# Autonomic control of the pulmonary circulation: Implications for pulmonary hypertension

**DOI:** 10.1113/EP092249

**Published:** 2024-10-25

**Authors:** Michael J. Plunkett, Julian F. R. Paton, James P. Fisher

**Affiliations:** ^1^ Department of Physiology, Faculty of Medical and Health Sciences, Manaaki Manawa – The Centre for Heart Research University of Auckland Auckland New Zealand

**Keywords:** baroreceptor, parasympathetic nervous system, pulmonary circulation, pulmonary hypertension, sympathetic nervous system

## Abstract

The autonomic regulation of the pulmonary vasculature has been under‐appreciated despite the presence of sympathetic and parasympathetic neural innervation and adrenergic and cholinergic receptors on pulmonary vessels. Recent clinical trials targeting this innervation have demonstrated promising effects in pulmonary hypertension, and in this context of reignited interest, we review autonomic pulmonary vascular regulation, its integration with other pulmonary vascular regulatory mechanisms, systemic homeostatic reflexes and their clinical relevance in pulmonary hypertension. The sympathetic and parasympathetic nervous systems can affect pulmonary vascular tone and pulmonary vascular stiffness. Local afferents in the pulmonary vasculature are activated by elevations in pressure and distension and lead to distinct pulmonary baroreflex responses, including pulmonary vasoconstriction, increased sympathetic outflow, systemic vasoconstriction and increased respiratory drive. Autonomic pulmonary vascular control interacts with, and potentially makes a functional contribution to, systemic homeostatic reflexes, such as the arterial baroreflex. New experimental therapeutic applications, including pulmonary artery denervation, pharmacological cholinergic potentiation, vagal nerve stimulation and carotid baroreflex stimulation, have shown some promise in the treatment of pulmonary hypertension.

## Abstract (highlights)

- **What is the topic of this review?**
  This review examines our understanding of the autonomic control of pulmonary circulation, with an emphasis on its clinical relevance and potential therapeutic targeting in pulmonary hypertension.
- **What advances does it highlight?**
  The sympathetic and parasympathetic nervous systems both regulate pulmonary vascular tone and stiffness, in integration with systemic autonomic homeostasis. Pulmonary vascular afferents responsive to pulmonary arterial pressure produce distinct pulmonary baroreflex responses. Dysfunction in autonomic control both to and from the pulmonary vasculature might contribute to pulmonary hypertension, and new approaches targeting this have demonstrated early success.

## 1 INTRODUCTION

The pulmonary circulation receives the entirety of the cardiac output (CO) from the right ventricle whilst maintaining low pressures and does so as a high‐flow, compliant, low‐resistance circuit (Barnes & Liu, 1995). Although the critical role of the autonomic control of the arterial circulation is well established (Fisher, Young et al., 2015; Thomas, 2011), the pulmonary vasculature is generally considered to be more influenced by passive haemodynamics, lung volume mechanics, respiratory gases, local endothelial regulators and humoral factors (Huertas et al., 2018; Suresh & Shimoda, 2016). However, the neural regulation of pulmonary vascular tone has recently become an area of reignited interest stimulated, in part, by the demonstration that therapeutic targeting of pulmonary innervation has clinical potential in the treatment of pulmonary hypertension (PH) (Zhang et al., 2022).

The pulmonary vasculature in humans and other mammalian species is innervated by a perivascular nerve plexus, with contributions from both catecholaminergic sympathetic nerve fibres, derived from the middle and inferior cervical ganglia and the first to fifth thoracic spinal ganglia, and parasympathetic, choline acetyltransferase‐positive fibres from the vagus nerve (Cavallotti et al., 2005; Haberberger et al., 1997; Kummer, 2011). This innervation is densest on the main pulmonary artery and becomes less extensive beyond the main pulmonary artery bifurcation towards the periphery, although species‐related differences exist in the extent to which these fibres innervate the intrapulmonary vascular bed (Barnes & Liu, 1995; Townsley, 2012). Pulmonary arteries and veins possess α‐ and β‐adrenergic and muscarinic receptors, with differences in distribution and subtypes between vessels and species (Barnes & Liu, 1995).

Despite its anatomical presence, the actions and functional role of the autonomic innervation of the pulmonary vasculature remain poorly understood and arguably under‐considered, particularly in human health and disease. This might be attributable, in part, to complexities in assessing pulmonary haemodynamics, with the gold‐standard right heart catheterization being an invasive procedure and with non‐invasive measurement by transthoracic echocardiography being indirect. In this context, the present review presents both classical insights and recent advances in our understanding of the autonomic control of the pulmonary circulation, with a particular focus on the clinical implications and applications in human PH.

## 2 NON‐NEURAL REGULATION OF THE PULMONARY VASCULATURE

Before outlining the current understanding of the neural regulation of the pulmonary vasculature, the important non‐neural regulators should briefly be acknowledged. Pulmonary vascular resistance (PVR) is strongly influenced by passive and mechanical factors. An increase in pulmonary blood flow (e.g., during exercise) leads to capillary recruitment, and following maximal recruitment, distension (Langleben et al., 2022), which reduces PVR (Kovacs et al., 2012). Lung volume also influences PVR but in a U‐shaped manner, with PVR being lowest around the functional residual capacity. Above functional residual capacity, PVR increases predominately owing to compression of alveolar vessels as alveolar pressure increases (Cheyne et al., 2020). When lung volume falls below functional residual capacity, PVR increases as extra‐alveolar vessels collapse owing to loss of elastic traction from lung parenchyma. Although changes in lung volume lead to large within‐breath modulation of sympathetic nerve activity (Plunkett, Holwerda et al., 2024), it is unclear whether this impacts the pulmonary vasculature directly.

Respiratory gases also have an important regulatory role in the pulmonary vasculature. Small intrapulmonary arteries respond to alveolar hypoxia with vasoconstriction via the local sensing of oxygen in pulmonary artery smooth muscle cells (Sommer et al., 2016). This response, known as hypoxic pulmonary vasoconstriction (HPV), is an essential mechanism to match pulmonary ventilation to perfusion (V˙/V˙Q˙Q˙ matching), although during global hypoxia generalized HPV leads to pulmonary hypertension. In addition, the pulmonary vasculature constricts in response to alveolar hypercapnia, which can also contribute to V˙/V˙Q˙Q˙ matching (Balanos et al., 2003). Finally, humoral and endothelium‐released vasodilatory regulators include nitric oxide (NO), prostacyclin, bradykinin, atrial natriuretic peptide, histamine and vasoactive intestinal peptide (Barnes & Liu, 1995; Suresh & Shimoda, 2016). The tonic release of NO from endothelial cells might be particularly important in the maintenance of basal low pulmonary vascular tone (Kiely et al., 1998; Stamler et al., 1994), and vasoconstrictor factors include endothelin, some arachidonic acid metabolites (i.e., prostaglandin E_2_, thromboxane and leukotrienes), serotonin and angiotensin II (Suresh & Shimoda, 2016).

## 3 SYMPATHETIC NERVOUS SYSTEM AND THE PULMONARY VASCULATURE

### 3.1 Animal studies

In early studies of intact anaesthetized cats and dogs, graded electrical stimulation of sympathetic nerves at the stellate ganglion was observed to cause graded pulmonary vasoconstriction (Hyman et al., 1981; Kadowitz & Hyman, 1973; Kadowitz et al., 1974). Vasoconstriction was mediated by noradrenaline acting on α_1_‐adrenergic receptors (Hyman & Kadowitz, 1985; Kadowitz & Hyman, 1973), although other neurotransmitters, such as neuropeptide Y and ATP, have since been implicated (Kummer, 2011). Pulmonary vasoconstriction occurs in both small pulmonary arteries and veins, with venocontraction potentially contributing up to half of the increase in PVR (Kadowitz et al., 1975). β_2_‐Adrenergic receptors are also innervated by sympathetic efferents, and when activated, cause pulmonary vasodilatation (Howard et al., 1975; Hyman et al., 1981), which appears to be mediated, at least in part, by endothelial NO production (Leblais et al., 2008).

A potential concern with these early studies is that general anaesthesia might have altered pulmonary vascular responses to adrenergic antagonism (Nyhan et al., 1989) and that examining responses at only a single blood flow might provide an incomplete picture, given the passive effects of changes in pulmonary blood flow. To address these concerns, Murray et al. (1986) measured pulmonary artery pressures across a range of CO, produced through graded constriction of the inferior vena cava, in conscious dogs. They confirmed the vasoconstrictive α‐adrenergic action and vasodilatory β‐adrenergic action with different pulmonary arterial flows. Combined α‐ and β‐adrenergic blockade was vasoconstrictive, suggesting that the net effect of tonic sympathetic activity was vasodilatory. This conclusion was in line with the earlier observation that administration of 6‐hydroxy‐dopamine (6‐OHDA), to induce chemical sympathectomy in anaesthetized dogs, also increased resting PVR (Hales & Westphal, 1979). It should be noted that Tucker (1979) did not show changes in PVR in anaesthetized dogs following 6‐OHDA; however, PVR was tested 48 h after 6‐OHDA, instead of acutely, without confirmation of the adequacy of sympathectomy, in contrast to Hales & Westphal (1979). From these studies in dogs, it can be concluded that the tonic sympathetic action on the pulmonary arteries is vasodilatory and would be consistent with the low resting tone of the pulmonary vasculature; however, changes in sympathetic tone that altered the balance of α‐ and β‐adrenergic activity, in addition to species differences, could modify this.

Pulmonary vascular stiffness is also influenced by the sympathetic nervous system. Electrical sympathetic nerve stimulation increases the stiffness of large pulmonary arteries (Brimioulle et al., 1999; Ingram et al., 1970; Piene, 1976). Increases in proximal arterial stiffness occurred at levels of electrical sympathetic stimulation that were insufficient to increase PVR (Pace, 1971), suggesting that stiffness could be regulated separately from tone. Although the physiological function of regulation of pulmonary vascular stiffness separately from resistance is not well defined, such adjustments by the sympathetic nervous system could alter the workload of the right ventricle (RV) by changing the pulsatile afterload on the RV (Wang & Chesler, 2011). Furthermore, increases in distensibility would increase pulmonary blood flow pulsatility, which, in turn, might have a number of physiological functions, including improved pulmonary capillary recruitment (Presson et al., 2002), improved oxygen exchange (Hauge & Nicolaysen, 1980) and reduced PVR (Raj et al., 1992).

### 3.2 Human studies

In human pulmonary artery segments, adrenergic activation evokes responses that are consistent with the aforementioned animal studies. α‐Adrenergic activation (i.e., by noradrenaline, phenylephrine and metaraminol) induces vasoconstriction, whereas β‐adrenergic stimulation by isoprenaline induces vasodilatation (Boe & Simonsson, 1980; Currigan et al., 2014). These actions are maintained in vivo. Systemic administration of an α‐adrenergic receptor blocker (phentolamine) reduced right heart catheter‐measured PVR in healthy individuals (Taylor et al., 1965), whereas intravenous β‐adrenergic receptor blockade (propranolol) increased PVR and reduced pulmonary vascular distensibility (i.e., increased stiffness) in healthy individuals (Hilty et al., 2024). These studies indicate the presence of both sympathetic vasoconstrictor and vasodilator tone.

**FIGURE 1:**
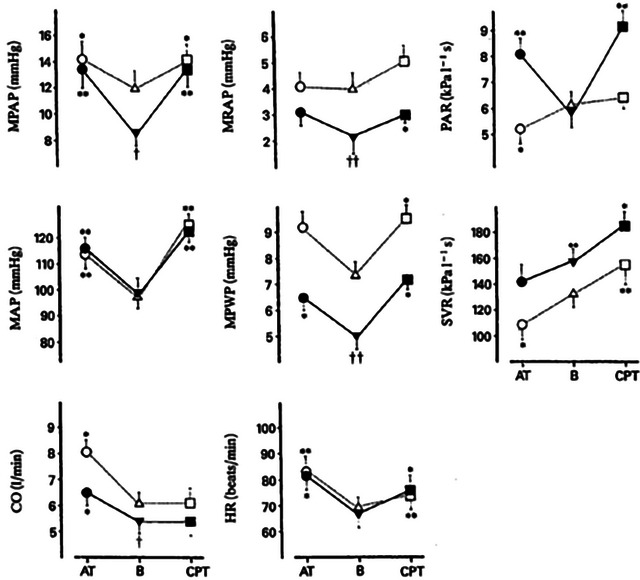
Responses to mental arithmetic (AT) and a cold pressor test (CPT) from baseline (B) in healthy humans, during control conditions (open symbols) and during inflation of an inferior vena cava balloon (filled symbols). Abbreviations: CO, cardiac output; HR, heart rate; MPAP, mean pulmonary arterial pressure; MPWP, mean pulmonary wedge pressure; SVR, systemic vascular resistance. ^*^ *P* < 0.05 and ^**^ *P* < 0.01 compared with baseline; ^†^ *P *< 0.05 and ^††^ *P* < 0.01 compared with control conditions. Reproduced with permission from Moruzzi et al. (1989a).

Given that it is not currently possible to stimulate or record selectively from the sympathetic nerves directed to the pulmonary vasculature in humans, sympatho‐excitatory manoeuvres have been used (e.g., mental stress, cold pressor test and exercise). Mental arithmetic increases mean pulmonary artery pressure (mPAP) secondary to increases in CO, whilst PVR falls, probably owing to passive vasodilatation and capillary recruitment (Figure 1) (Moruzzi et al., 1989a). With cold pressor testing, PVR remains unchanged, although left atrial pressure rises, increasing mPAP (Moruzzi et al., 1989a). Likewise, direct adrenergic stimulation by noradrenaline infusion increases mPAP without changing PVR (Hanson et al., 1973). When venous return to the heart is impeded (i.e., by inferior vena cava balloon or simulated haemorrhage) to blunt increases in CO, an increase in PVR is revealed during mental arithmetic, the cold pressor test and noradrenaline infusion (Hanson et al., 1973; Moruzzi et al., 1989a). Collectively, such findings indicate that sympathetically mediated pulmonary vasoconstriction could restrain excessive vasodilatation of the compliant pulmonary vasculature when CO is increased; however, passive flow‐related dynamics are the predominant influence.

Exercise is a powerful sympathetic driver, increasing CO and facilitating the redistribution of blood flow to active skeletal muscles by evoking vasoconstriction in less active regions (e.g., splanchnic and renal circulations) (Fisher, Young et al., 2015). Pulmonary vasoconstriction might be expected; however, decreases in PVR are observed (Kovacs et al., 2012), largely attributable to passive flow‐related vascular recruitment and dilatation (Naeije & Chesler, 2012). Invasive monitoring of pulmonary haemodynamics in healthy humans during exercise with selective α‐ and β‐adrenergic blockade has not been undertaken, but such investigations have been conducted in sheep performing strenuous treadmill exercise (Kane et al., 1993). In control conditions, PVR reduced with exercise and was not reduced further with α‐adrenergic blockade, potentially representing a maximally dilated pulmonary vasculature. In contrast, β‐blockade increased PVR compared with control conditions such that the normal reduction in PVR was lost, suggesting that β‐adrenergic activity exerts a restraining influence on α‐adrenergic vasoconstriction. Indeed, during combined adrenergic blockade, no net effect of sympathetic activation on the pulmonary vasculature was observed. In support of this concept, labetalol, a combined α‐ and β‐adrenergic antagonist with greater β‐adrenergic potency, increased the PVR index during exercise in essential hypertension (Koch, 1977; Svendsen et al., 1980).

**FIGURE 2:**
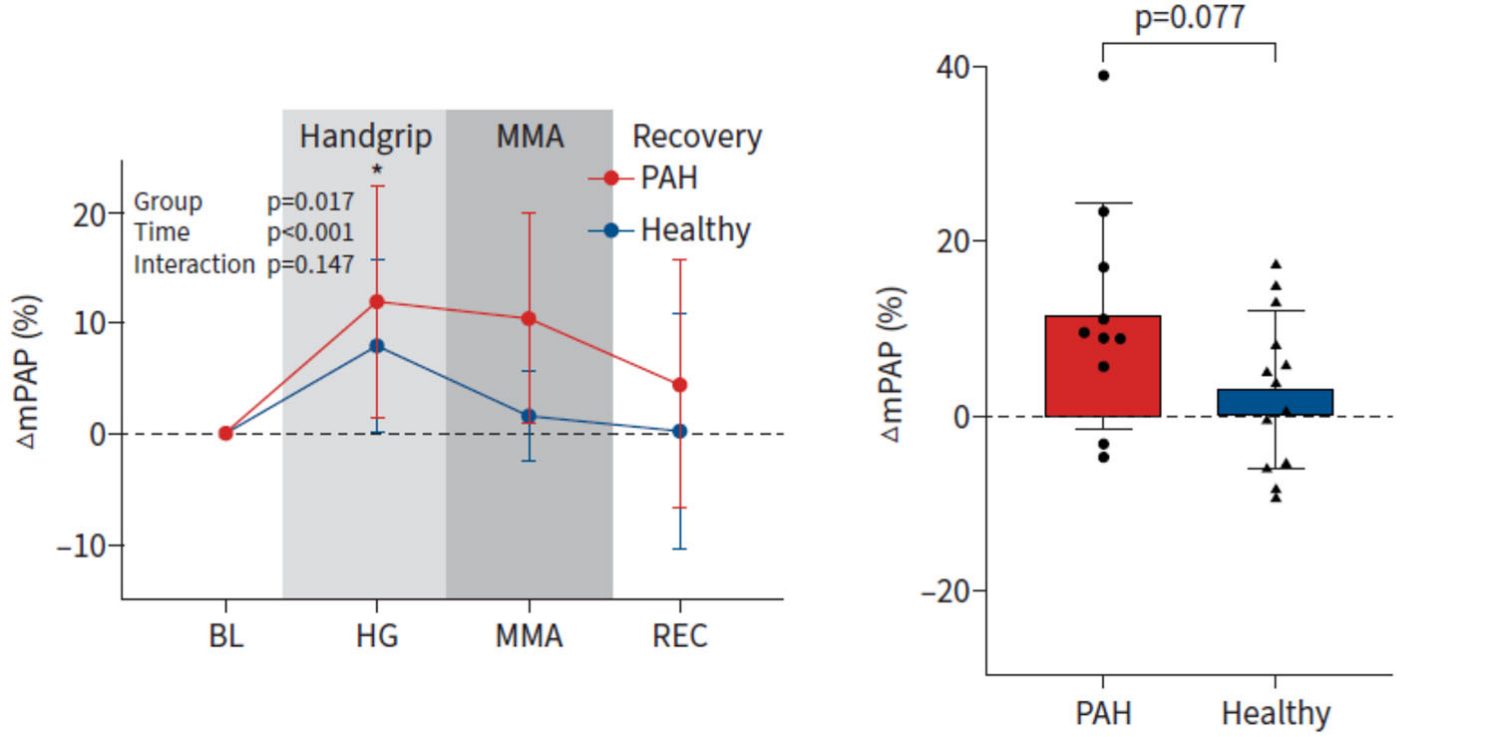
In the left panel, the percentage change in mean pulmonary artery pressure (∆mPAP) from baseline (BL) is shown with isometric handgrip exercise (HG), followed by muscle metaboreflex activation (MMA) with postexercise circulatory occlusion (PECO), then recovery (REC), in pulmonary arterial hypertension patients (PAH; red) compared with healthy control participants (blue). In the right panel, the muscle metaboreflex‐induced response in mPAP (defined as ∆mPAP during MMA minus ∆mPAP during a free‐flow recovery without PECO) is shown in PAH patients and healthy control subjects. *n* = 10 PAH patients and *n* = 14 healthy control participants. Values are shown as the means ± SD. ^*^ *P *< 0.05 PAH compared with healthy control subjects. Reproduced from Plunkett, Sayegh et al. (2024) with permission of the ERS 2024: *European Respiratory Journal*, *63*(1), 2300952; https://doi.org/10.1183/13993003.00952‐2023 Published 4 January 2024.

Activation of metabolically sensitive skeletal muscle afferents is an important mechanism whereby exercise‐induced sympathetic excitation occurs (i.e., muscle metaboreflex) (Fisher, Fernandes et al., 2015). The isolated activation of the muscle metaboreflex with postexercise circulatory occlusion in healthy humans evokes an increase in pulmonary artery systolic pressure (PASP), despite the return of CO to baseline, indicative of pulmonary vasoconstriction (Lykidis et al., 2008). In chronic disease conditions, in which the muscle metaboreflex can be exaggerated, muscle metaboreflex activation could induce excessive pulmonary vasoconstriction and impede the CO response. Testing this notion in pulmonary arterial hypertension (PAH), we demonstrated that isolated muscle metaboreflex activation leads to augmented pulmonary vasoconstriction, because pulmonary artery pressure increased without a parallel increase in CO, which was not observed in healthy control subjects (Figure 2) (Plunkett, Sayegh et al., 2024).

### 3.3 Clinical significance

The chronic activation of the sympathetic nervous system has been identified in a number of clinical conditions and is associated with poor pathophysiological and clinical outcomes (Fisher & Paton, 2012; Fisher et al., 2009), including in PAH (Ciarka et al., 2010; Velez‐Roa et al., 2004). Such heightened sympathetic activation could exacerbate PH through α‐adrenergic vasoconstriction (Salvi, 1999) and increases in stiffness inducing higher pulsatile RV afterload. Small experimental studies using α‐adrenergic blockade have reported reductions in mPAP and PVR in PH in children (Grover et al., 1961), altitude‐related PH (Hackett et al., 1992) and PH secondary to chronic obstructive pulmonary disease (Lewczuk et al., 1990), although not in PAH (Hermiller et al., 1982). Larger clinical trials of adrenergic antagonism in PAH have focused on β‐adrenergic blockade, based on its clinical efficacy in heart failure. Unfortunately, outcomes of β‐adrenergic blockade in PAH are disappointing (Vaillancourt et al., 2017) and include findings of worsened exercise capacity (van Campen et al., 2016), in addition to worsened exercise capacity and PVR in portopulmonary hypertension (Provencher et al., 2006). This might be attributable, at least in part, to interference with α‐ and β‐adrenergic balance, with the consequence of β‐blockade being impaired restraint of α‐adrenergic vasoconstriction. In contrast, potentiation of β‐adrenergic pulmonary vasodilatation using albuterol, an inhaled selective β_2_‐agonist, improved exercise PVR and pulmonary arterial compliance in patients with heart failure with preserved ejection fraction (Reddy et al., 2019).

Increased pulmonary vasoconstriction in response to sympathetic activation has been hypothesized to be a contributory driver to steepened mPAP–CO relationships in conditions such as PAH and left ventricular heart failure, in addition to impaired pulmonary vascular recruitability and distensibility (Lewis et al., 2013). In PAH, the mPAP–CO relationship was steeper during exercise in comparison to flow increases induced by dobutamine, supporting the involvement of non‐passive factors, including increased sympathetic vasoconstriction (Kafi et al., 1998). People with essential hypertension exhibit increases in PVR during mental arithmetic and cold pressor testing not observed in healthy individuals (Fiorentini et al., 1985; Moruzzi et al., 1989b), demonstrating that augmented pulmonary vascular sympathetic reactivity can occur in disease. The mechanism by which this occurs (i.e., a greater sympathetic outflow response to the pulmonary vasculature, a greater vasoconstrictive response to the sympathetic stimulus, or both) remains unknown. Furthermore, whether increased pulmonary vascular reactivity to sympathetic stressors contributes to PH either at rest or to exercise is a proposition that remains untested.

## 4 PARASYMPATHETIC NERVOUS SYSTEM AND THE PULMONARY VASCULATURE

### 4.1 Animal studies

In species with undivided pulmonary and systemic circulatory systems (i.e., most reptiles and amphibians) parasympathetic stimulation induces potent pulmonary vasoconstriction (Filogonio et al., 2020) and controls shunting between pulmonary and systemic circulations based upon metabolic demands (Burggren et al., 2020). For example, in the freshwater turtle, efferent vagal regulation of pulmonary vasculature tone matches pulmonary blood flow to ventilation (cardiorespiratory synchrony) by altering shunting to optimize oxygen uptake (Wang & Hicks, 1996). In mammalian species, however, the functional significance of parasympathetic pulmonary vascular control remains uncertain.

Stimulation of cervical vagus nerves and acetylcholine infusion cause pulmonary vasoconstriction (Matran et al., 1991; Nandiwada et al., 1983; Sada et al., 1987). However, rather than representing a pure parasympathetic action, vasoconstriction could be mediated by sympathetic fibres within the vagus nerve (Onkka et al., 2013) or stimulation of sympathetic nerve activity via the arterial baroreflex as a response to vagally mediated systemic hypotension (Matran et al., 1991). There could be species differences in the responses of pulmonary veins, because acetylcholine did cause venoconstriction in dogs (Barman et al., 1989; Hyman, 1969), whereas in rabbits vagal nerve stimulation had no effect (Sada et al., 1987).

To assess cholinergic action on the pulmonary vasculature directly, without confounding systemic circulatory influences, Barman et al. (1989) infused acetylcholine into the haemodynamically isolated pulmonary vascular circuit of dogs under constant‐flow perfusion and measured the responses of both pulmonary arteries and veins via fine intravascular catheters. In large arteries, vasodilatation occurred at low doses of acetylcholine, whereas in small and large pulmonary veins venoconstriction occurred at high doses. An overall increase in pulmonary resistance was seen only with the highest dose. However, when the pulmonary vascular tone is already elevated, the response to vagal nerve stimulation and acetylcholine infusion is usually vasodilatory (McMahon et al., 1992; Nandiwada et al., 1983) and mediated by the endothelium and NO (Barman et al., 1989; McMahon et al., 1992), as seen in the brain (Iadecola & Zhang, 1996).

### 4.2 Human studies

In human pulmonary arterial segments with resting tone, acetylcholine causes vasoconstriction, mediated by muscarinic M_3_ receptors in vascular smooth muscle (Walch et al., 1997). When precontracted by noradrenaline, acetylcholine vasodilates human pulmonary arterial segments, mediated by muscarinic M_1_ and M_3_ receptors (Currigan et al., 2014; Norel et al., 1996; Walch et al., 2000). As in animals, the vasodilatory responses to acetylcholine are mediated by the endothelial release of NO and prostaglandins (Norel et al., 1996; Zhang et al., 1996). In pulmonary vein segments with resting tone, acetylcholine causes slight vasodilatation, and more marked vasodilatation when precontracted, with responses mediated by endothelial muscarinic M_1_ receptors (Walch et al., 2000, 1997). Acetylcholine infusion directly into the pulmonary artery or right atrium in healthy individuals reduces PVR, a response further enhanced when the resting tone is increased during hypoxia (Conraads et al., 1994; Fritts et al., 1958). In summary, pulmonary vascular responses to parasympathetic stimulation are complex, with both vasoconstricting and vasodilatory effects, and the degree of stimulus, tone and type of vessel in the pulmonary vasculature all appear to be important but remain incompletely understood.

In comparison to the effects of its stimulation, less is known about the tonic parasympathetic action on the pulmonary circulation in humans. Hilty et al. (2024), in healthy individuals, compared the effects of dual systemic muscarinic and β‐adrenergic blockade, using glycopyrrolate and propranolol, with β‐adrenergic blockade alone, on resting pulmonary haemodynamics and pulmonary vascular distensibility measured through thigh‐cuff‐release manoeuvres. The addition of muscarinic blockade ameliorated the increase in resting PVR with β‐adrenergic blockade. However, CO increased, which could have mediated this effect passively, independent of actions on vascular tone. Combined muscarinic and β‐adrenergic blockade reduced pulmonary vascular distensibility more than β‐adrenergic blockade alone, implicating cholinergic activity in the regulation of pulmonary vascular stiffness.

Parasympathetic innervation of the pulmonary vasculature might have a functional role in regulating pulmonary blood volume (PBV). In humans, exaggerated increases in PBV occurred in response to central volume expansion by intravenous dextran infusion with muscarinic blockade, attributed to large increases in pulmonary vascular distensibility (+518%) and reductions in PVR (−32%) (Giuntini et al., 1966). This could be important in maintaining the stability of PBV, such as during exercise, when CO, thoracic and cardiac blood volumes all increase but PBV remains stable (Iskandrian et al., 1982). Increases in PBV could reduce lung compliance (Hauge et al., 1975) and might increase the work of breathing.

### 4.3 Clinical significance

Pulmonary arterial acetylcholinesterase activity is reduced in PAH (da Silva Goncalves Bos et al., 2018), possibly as an adaptive upregulated vagal postganglionic response to an impairment of vagal function at or proximal to the level of the ganglion, similar to heart failure (Bibevski & Dunlap, 2011). In a rat model of PAH, chronic pyridostigmine‐mediated potentiation of parasympathetic activity and vagal nerve stimulation both improved histological features of pulmonary arterial remodelling, such as reduced pulmonary wall thickness and occlusive vascular lesion formation, and reduced PVR (da Silva Goncalves Bos et al., 2018; Yoshida et al., 2018). Acutely, however, vagal nerve stimulation in rats with PAH did not reduce either mPAP or PVR, despite exerting a cardiovagal response with reduced HR, suggesting that improvements did not occur owing to a relaxation of pulmonary vascular tone (Yoshida et al., 2018). Endothelial dysfunction in PAH could impair vasodilatory responses to vagal/cholinergic stimulation, and in fact, acetylcholine has been reported to cause pulmonary vasoconstriction in two PAH patients (Conraads et al., 1994). Thus, although parasympathetic potentiation shows some promise from preclinical studies in the form of reducing the remodelling of the pulmonary artery and RV in PAH, whether its vasodilatory actions can be targeted therapeutically remains unclear and is potentially limited by its endothelial dependence in conditions, such as PAH, that are characterized by endothelial dysfunction.

## 5 PULMONARY BARORECEPTORS

### 5.1 Autonomic afferents arising from the pulmonary vasculature

**FIGURE 3:**
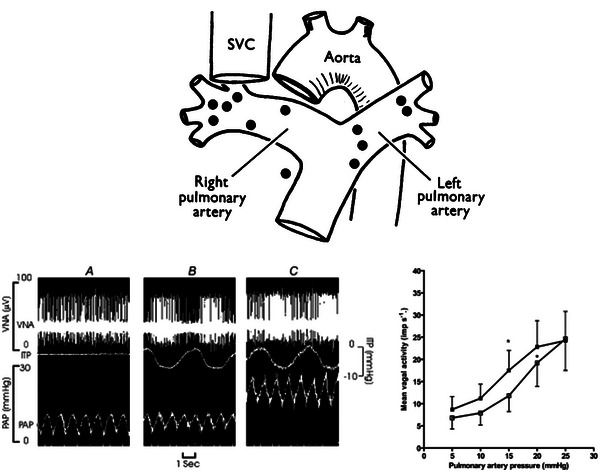
The top panel shows the location of vagal afferent pulmonary arterial baroreceptors as determined by electrophysiological testing in dogs. Receptors were located predominately in the main pulmonary artery, its bifurcation and the proximal right and left pulmonary artery branches. Reproduced with permission from Coleridge & Kidd (1960). Abbreviation: SVC, superior vena cava. The bottom panels depict pulmonary baroreceptor vagal nerve activity (VNA) in close‐chested dogs with a vascularly isolated pulmonary circulation, reproduced with permission from Moore et al. (2004b). In the left panel, an original pulmonary baroreceptor VNA recording is shown from one dog, with pulsations of pulmonary artery pressure (PAP) without negative phasic intrathoracic pressure (ITP; *A*), VNA with negative phasic ITP (*B*) and increased VNA with a step increase in PAP with negative phasic ITP (*C*). The right panel shows the mean VNA with increases in PAP at non‐phasic atmospheric ITP (open squares) and negative phasic ITP (filled squares). The mean threshold PAP for VNA response reduced from 12 to 9.5 mmHg with negative phasic ITP. ^*^ *P *< 0.05 compared with baseline VNA.

Early electrophysiological work in animals identified the existence of receptors located near the area of the bifurcation of the main pulmonary artery trunk into the left and right pulmonary arteries (Figure 3) that were innervated by myelinated vagal nerve fibres whose discharge was synchronized with the pulmonary arterial pulse and was increased with pulmonary arterial pressure (Coleridge & Kidd, 1960; Coleridge et al., 1961; Pearce & Whitteridge, 1951). Sympathetic pulmonary arterial afferents, myelinated and unmyelinated, responsive to pressure have been identified in cats, with endings in the left pulmonary artery (Nishi et al., 1974), and in dogs, in the main pulmonary artery trunk (Uchida, 1975). Sympathetic afferent discharge increased in synchrony with the pulmonary systolic pulse when pulmonary arterial pressure was increased above a threshold. However, this occurred only transiently and was not increased further with additional increases in pressure (Nishi et al., 1974). This contrasts with vagal afferents that show sustained increases in activity with step increases in pressure (Moore et al., 2004b).

Pulmonary artery distension with an inflatable cuff in dogs produced a reflex pulmonary vasoconstriction and increase in pulmonary artery pressure (Osorio & Russek, 1962). This positive feedback ‘pulmo‐pulmonary baroreflex’ has been confirmed in a number of other investigations, including in conscious animals (Hyman, 1968; Juratsch et al., 1980; Laks et al., 1975; Ueda et al., 1965), with reflexive vasoconstriction identified in both arteries and veins (Hyman, 1968), in contrast to the negative feedback responses of the arterial baroreflex. A further effect of this reflex was a reduction in pulmonary blood volume (Hyman, 1968). The pulmo‐pulmonary reflex is mediated by neural afferents, because it is abolished by denervation or local anaesthesia of the distended pulmonary artery. These might be sympathetic pulmonary arterial afferents rather than vagal afferents, because the reflex is unaffected by bilateral vagotomy (Aramendia et al., 1963; Hyman, 1968; Juratsch et al., 1980; Osorio & Russek, 1962; Ueda et al., 1965). Sympathetic nerve fibres might also mediate the efferent path of this reflex, because vasoconstriction was abolished by chemical sympathetic denervation with 6‐OHDA, which selectively destroys adrenergic nerve terminals, in conscious dogs (Juratsch et al., 1980). However, sympathetic mediation of this reflex was not confirmed in other studies (Aramendia et al., 1963; Hyman, 1968; Ueda et al., 1965). These discrepant findings could relate to general anaesthesia and the differential efficacy of methods used to disrupt sympathetic nerve transmission. Aramendia et al. (1963) and Hyman (1968) used intraperitoneal reserpine, which blocks vesicular monoamine transporters, depleting peripheral sympathetic nerves of noradrenaline, which, however, leaves purinergic sympathetic transmission intact, in contrast to 6‐OHDA, which disrupts both noradrenergic and purinergic transmission (Warland & Burnstock, 1987). Aramendia et al. (1963) performed sympathectomy by excision of the thoracic ganglion, which could leave some sympathetic innervation intact via cervical ganglia, and Ueda et al. (1965) did not describe their method of sympathectomy.

Studies using vascularly isolated, perfused pulmonary artery preparations demonstrated that experimental elevations in pulmonary arterial pressure (i.e., pulmonary baroreceptor activation) caused sympathetic efferent‐mediated increases in systemic vascular resistance and arterial pressure (Kan et al., 1979; Ledsome et al., 1981; McMahon et al., 2000; Moore et al., 2011) and increased activity in vagal afferents arising from pulmonary baroreceptors (Figure 3) (Moore et al., 2004b). Furthermore, increasing pulmonary artery pressure also stimulated phrenic nerve and diaphragm activity (Kan et al., 1979; Ledsome et al., 1981; McMahon et al., 2000). These reflex responses were consistently prevented by vagotomy and, as such, mediated by vagal pulmonary arterial afferents. Application of negative phasic intrathoracic pressure to mimic respiration lowered the threshold of pulmonary arterial pressure required to elicit systemic vascular (mPAP of 20 mmHg) (Moore et al., 2004a) and vagal afferent nerve activity responses (mPAP of 15 mmHg) (Moore et al., 2004b) to within the physiological range of pulmonary arterial pressure, demonstrating pulmonary baroreflex engagement in physiologic conditions.

The first evidence in humans for the existence of a pulmonary baroreflex was provided by Simpson et al. (2020). Thirteen healthy lowlanders were studied at high altitudes, in order to potentiate baseline pulmonary artery pressure (PASP mildly elevated at 32 mmHg) and pulmonary baroreceptor activation. Muscle sympathetic nerve activity was measured before and after selective pulmonary arterial vasodilatation by inhaled NO. The PASP was reduced by ∼20% and, in parallel, muscle sympathetic nerve activity by ∼25%. Reductions in pulmonary artery pressure also reset the systemic arterial baroreflex to lower resting diastolic blood pressure and muscle sympathetic nerve activity.

### 5.2 Physiological significance

**FIGURE 4:**
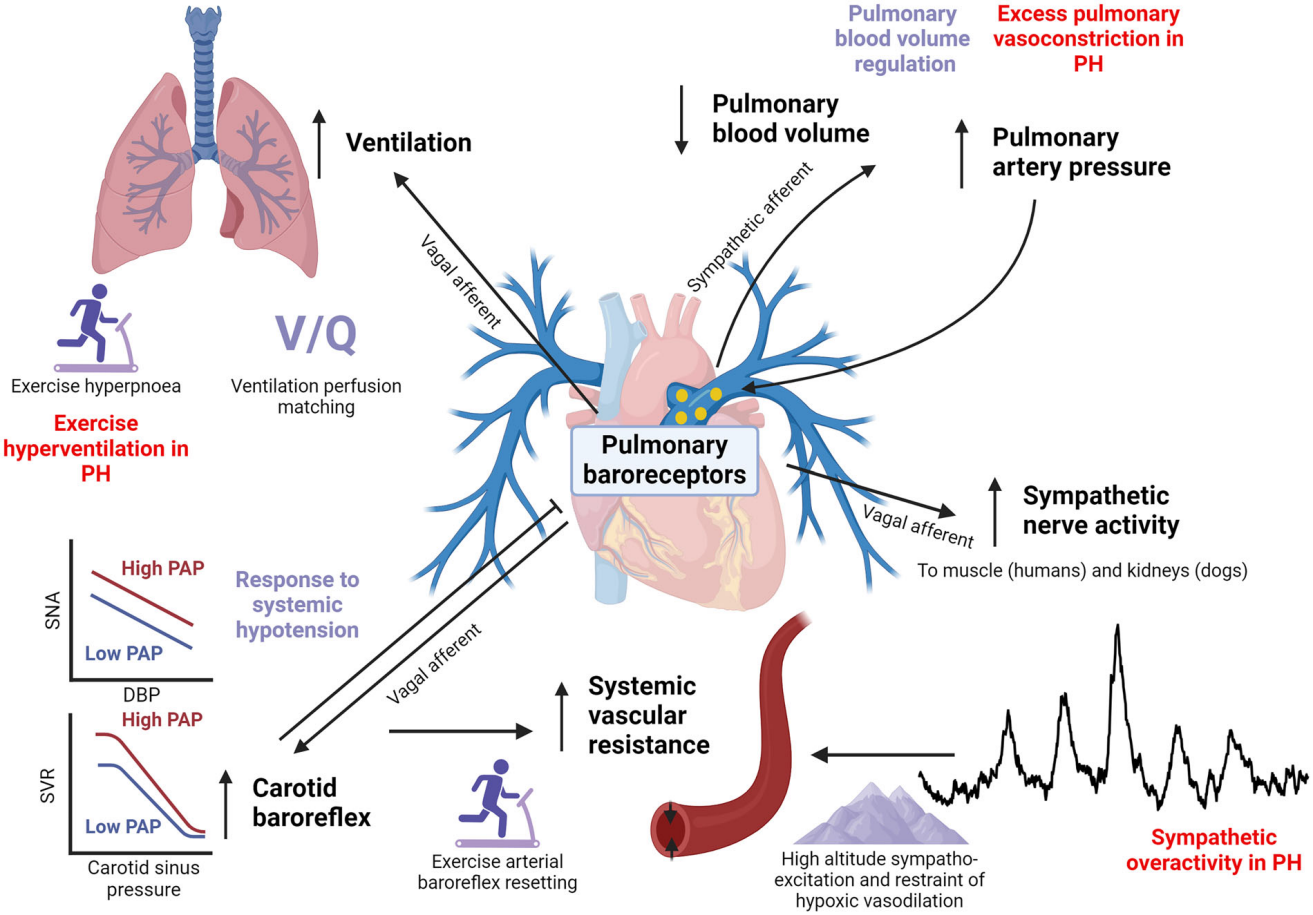
Summary of the known actions of pulmonary baroreceptors, their potential physiological function (purple) and significance in pulmonary hypertension (PH; red). Pulmonary baroreceptors increase their discharge in response to increases in pulmonary arterial pressure and distension. This produces a feed‐forward pulmo‐pulmonary reflex, probably mediated by sympathetic pulmonary arterial afferents, that increases pulmonary vasoconstriction and pressure and reduces pulmonary blood volume, which might have a role in regulating pulmonary blood volume but might also perpetuate pulmonary vasoconstriction in PH. Activation of the pulmonary baroreflex increases sympathetic nerve outflow (to skeletal muscle and kidneys) mediated by vagal pulmonary arterial afferents, which could be responsible, in part, for increased sympathetic activity at high altitude and for sympathetic overactivity in PH. Pulmonary baroreflex activation shifts the carotid baroreflex to higher blood pressure and sympathetic outflow (SNA), probably mediated by vagal afferents, which contributes to an increase in systemic vascular resistance (SVR) and arterial pressure. Conversely, carotid baroreflex activation inhibits the pulmonary baroreflex such that sympathetic outflow and systemic blood pressures are lower at a given pulmonary artery pressure. These interactions between pulmonary and systemic baroreflexes could have a role in cardiorespiratory exercise responses, responses to systemic hypotension and restraint of peripheral hypoxic vasodilatation at high altitudes. Finally, pulmonary baroreflex activation stimulates phrenic nerve activity and increases respiratory drive mediated by vagal afferents, which might have a role in the generation of exercise hyperpnoea and could optimize ventilation‐to‐perfusion matching (V˙/V˙Q˙Q˙) by increasing ventilation when pulmonary blood flow increases distension of pulmonary arteries. Created in BioRender. Plunkett, M. (2024) BioRender.com/i27k675.

Despite animal and human evidence supporting a distinct pulmonary baroreflex, responsive to increases in pulmonary arterial distension and pressure, that increases pulmonary vasoconstriction, probably via sympathetic pulmonary arterial afferents, and increases sympathetic outflow, systemic vascular resistance and respiratory drive via vagal pulmonary arterial afferents (Figure 4), its physiological significance is not well established. Reflexive increases in pulmonary vasomotor tone in response to pulmonary arterial distension could protect the pulmonary vasculature from unrestrained increases in pulmonary volume and vasodilatation when pulmonary blood flow increases, such as with exercise. Increases in ventilation with increased pulmonary distension might aid in matching pulmonary blood flow to ventilation. The pulmonary baroreflex might also contribute to systemic cardiorespiratory adjustments in exercise, whereby increased pulmonary arterial flow and transmural pressure exerted by exercise hyperpnoea activate the pulmonary baroreflex, leading to increased systemic vasoconstriction, upwards resetting of the arterial baroreflex and increased respiratory drive in a positive feedback manner (Hainsworth, 2014; McMahon et al., 2000). At high altitudes, the pulmonary baroreflex might increase sympathetic nerve activity and restrain systemic hypoxic vasodilatation (Simpson et al., 2020). Potential consequences of this reflex could emerge in PH, where increased pulmonary baroreflex activation could increase sympathetic nerve activity (Hainsworth, 2014), exacerbate exercise hyperventilation (Weatherald et al., 2020) and lead to positive feedback pulmonary vasoconstriction via the pulmo‐pulmonary baroreflex.

### 5.3 Clinical significance

Percutaneous catheter‐based ablation in the pulmonary artery, termed pulmonary artery denervation (PADN) (Chen, Zhang, Xu et al., 2013), aims to interrupt the sympathetic nervous innervation of the pulmonary artery and/or the pulmonary baroreflex and thereby reduce pulmonary artery pressure and resistance in PH. In animal models, PADN acutely and progressively reduces sympathetic nerve conduction velocity, myelin sheath thickness, axonal diameter and the density of pulmonary artery sympathetic nerve fibres (Jiang et al., 2020; Rothman et al., 2019; Zhou et al., 2015). PADN disrupted the pulmo‐pulmonary baroreflex in dogs (Chen, Zhang, Zhou et al., 2013) but also blunted the response to pulmonary vasoconstrictor infusion in swine (Rothman et al., 2019). In addition, PADN reduces both pulmonary artery and RV remodelling, with downregulation of renin–angiotensin–aldosterone, noradrenergic and neuropeptide Y signalling (Huang et al., 2019; Liu et al., 2016).

In a first‐in‐human study, PADN in PAH patients improved mPAP, pulmonary arterial compliance and PVR at 3 months of follow‐up (Chen, Zhang, Xu et al., 2013). The improvement in PVR was also noted immediately after PADN, supporting disruption of neurally mediated pulmonary vasoconstriction. Following this, a single‐blinded randomized sham‐controlled trial of PADN was conducted with 128 treatment‐naïve PAH patients (Zhang et al., 2022). At 6 months follow‐up, PVR was reduced by 27%, in comparison to 15% with sham, and 6‐min walk distance increased by 34 m above sham, whilst RV functional measures improved. PADN also improved PVR and 6‐min walk distance in other sham‐controlled randomized trials of chronic thromboembolic PH (Romanov et al., 2020) and combined pre‐ and postcapillary PH associated with left heart disease (Zhang et al., 2019).

## 6 SYSTEMIC AND PULMONARY BAROREFLEX INTERACTIONS

**FIGURE 5:**
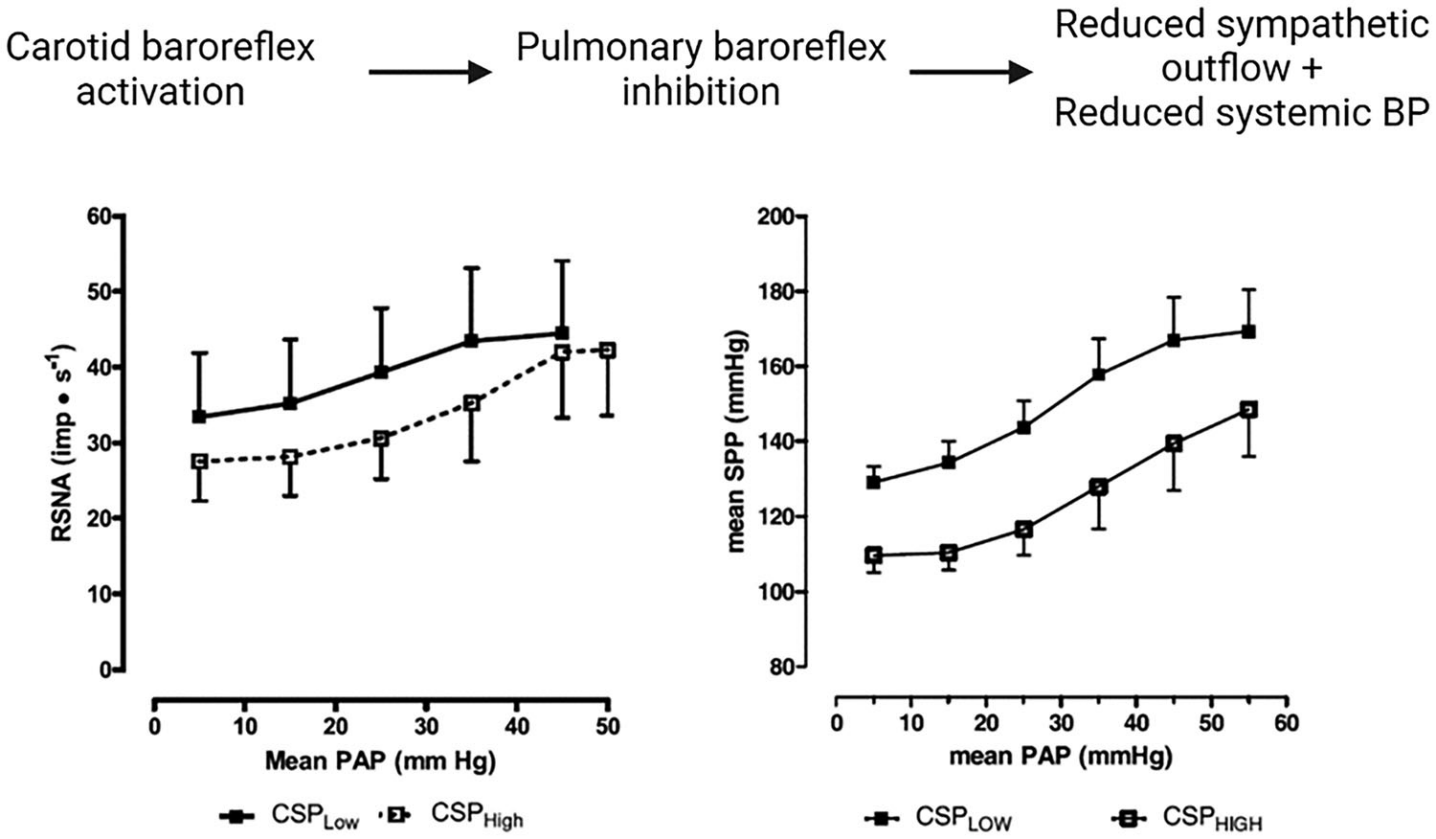
The schematic diagram at the top depicts the effect of carotid baroreflex activation (i.e., higher carotid sinus pressure; CSP) in inhibiting pulmonary baroreflex responses, reducing sympathetic outflow and systemic blood pressure to a given pulmonary arterial pressure (PAP). Depicted in the graphs, in closed‐chested dogs with vascularly isolated pulmonary circulation, Moore et al. (2011) examined the influence of low (60 mmHg) and high (126 mmHg) CSP on renal sympathetic nerve activity (RSNA; left panel) and systemic perfusion pressure (SPP; right panel) responses to increased pulmonary baroreceptor activation by increasing isolated PAP. Values are means ± SEM. Reproduced with permission.

Given the interconnected nature of the systemic and pulmonary circulations, it is unsurprising that systemic mechanisms for maintaining vascular homeostasis should influence pulmonary vasculature control. Arterial baroreceptor unloading (i.e., reducing systemic arterial pressure) increases PVR, stiffens the proximal pulmonary vasculature, reduces pulmonary vascular capacitance and steepens the pulmonary artery pressure–flow relationship in dogs, via α‐adrenergic receptor activation (Brimioulle et al., 1999; Peterson et al., 1993). Collectively, this response appears to restore impairments in the efficiency of RV work induced by the reduced CO (Brimioulle et al., 1999). Carotid baroreflex activation resets the pulmonary baroreflex to a lower systemic pressure and renal sympathetic nerve activity set point (Figure 5) (Moore et al., 2011). Such systemic baroreflex and pulmonary vascular interactions might have functional significance in responses to systemic hypotension. Initially, arterial baroreflex unloading would lead to sympatho‐excitation supported by enhanced pulmonary baroreceptor sympathetic outflow, reset to increased activation at lower pulmonary pressures. Subsequent increases in PVR and reduced pulmonary vascular capacitance could redistribute blood from the pulmonary circulation to support systemic blood pressure. Using a novel approach, Wang et al. (2024) observed that chronic carotid baroreceptor stimulation in a rat PAH model reduced pulmonary artery sympathetic nerve density, PVR, mPAP and pulmonary artery remodelling after 4 weeks. Whether the manipulation of such carotid baroreceptor and pulmonary vascular interactions has a potential therapeutic role in the treatment of PH is an interesting proposition, because carotid stimulator devices have already been developed and trialled in human essential hypertension (Heusser et al., 2016), although effects on systemic blood pressure regulation in PH would need consideration.

## 7 SYSTEMIC HYPOXIA AND AUTONOMIC CONTROL OF THE PULMONARY CIRCULATION

Systemic hypoxia causes autonomic adjustments (Fisher et al., 2018; Siebenmann et al., 2019; Simpson et al., 2024), which, given the autonomic influences on the pulmonary vasculature outlined earlier in this review, could lead to an autonomic modulation of the local HPV response. Several studies assessing autonomic modulation of HPV in response to acute hypoxia have been performed in animals. α‐Adrenoreceptor activation enhances the HPV in dogs (Brimioulle et al., 1997; Olson et al., 1982), whereas β‐adrenoreceptor activation (Brimioulle et al., 1997; Shirai et al., 1994) attenuates the HPV in dogs and cats. However, vagotomy and atropine had no effect on HPV in dogs (Lejeune et al., 1989). Combined α‐ and β‐adrenergic blockade augments the HPV in anaesthetized dogs and cats (Brimioulle et al., 1997; Shirai et al., 1994), suggesting that, on balance, sympathetic activation attenuates the HPV during acute systemic hypoxia. However, this has not been demonstrated consistently. In earlier studies, α‐ and β‐adrenergic blockade in conscious dogs were found not to change HPV (Lodato et al., 1988), and studies of chemical sympathectomy using 6‐OHDA also found no alteration in HPV (Hales & Westphal, 1979; Naeije et al., 1989). Likewise, studies assessing the effects of combined sympathetic and parasympathetic blockade during acute hypoxia have also demonstrated inconsistent findings. Shirai et al. (1994) observed in cats that the HPV‐induced reduction in small pulmonary vessel internal diameter was greatly enhanced by autonomic ganglionic blockade with hexamethonium, a nicotinic receptor antagonist, combined with bilateral adrenalectomy. However, in dogs, hexamethonium (without adrenalectomy) had no effect on HPV (Lodato et al., 1988). Although adrenalectomy might have prevented the action of circulating catecholamines on the pulmonary vasculature, given that Lodato et al. (1988) also observed no difference with combined α‐ and β‐adrenergic blockade, this does not seem to explain the discrepancies. Taken together, it appears that some animal studies suggest an attenuating role of the autonomic nervous system on HPV, but there are discrepancies between studies, and the reasons for this remain unresolved.

In humans, changes in PASP, measured by transthoracic echocardiography, in response to acute hypoxia were recorded before and after combined sympathetic and parasympathetic blockade with trimethaphan, which interrupts cholinergic transmission in autonomic ganglia (Liu et al., 2007). Autonomic blockade blunted pulmonary vascular responses to acute hypoxia. However, given that autonomic blockade prevented the normal increase in CO in response to hypoxia, Liu et al. (2007) adjusted PASP responses for this blunting of CO response based upon previously published CO–pulmonary artery pressure relationships (Balanos et al., 2005). After such adjustment, they suggested that there was no effect of autonomic blockade on pulmonary vascular responses during acute hypoxia. However, given that the effect of changes in CO on pulmonary artery pressure was not examined directly in this study, the possibility cannot be excluded that a true autonomic attenuation of HPV was observed.

Chronic exposure to hypoxia (i.e. in the setting of acclimatization to high altitude) leads to more profound increases in sympatho‐excitation (Fisher et al., 2018), which could result in altered autonomic influences on HPV during chronic hypoxia in comparison to acute hypoxia. Liu et al. (2007) also studied the pulmonary vascular responses to acute hypoxia, 30 min after recovery from an 8 h period of sustained hypoxia to simulate early acclimatization, with and without trimethaphan. Autonomic blockade following the sustained hypoxic exposure blunted HPV, but this effect was not observed after adjustment for CO, as described above for acute hypoxia alone. In healthy lowlanders, Hilty et al. (2024) determined HPV by comparing right heart catheter‐measured PVR at sea level and at high altitude (3‐week exposure), with and without β‐adrenergic blockade. β‐Adrenergic blockade augmented HPV, suggesting an attenuation of HPV by β‐adrenergically mediated vasodilatation. This effect was no longer present when β‐adrenergic blockade was combined with muscarinic blockade, potentially indicative of a cholinergic potentiation of HPV, but the contribution of drug‐induced changes in CO cannot be ruled out (Hilty et al., 2024). Importantly, α‐adrenergic blockade did not lower PVR in healthy individuals at high altitudes but did lower PVR in individuals with high‐altitude pulmonary oedema (Hackett et al., 1992).

Although an autonomic attenuation of HPV in the setting of sustained global hypoxia would have a plausible physiological function in limiting the development of PH, the overall contribution of autonomic responses during systemic hypoxia remains incompletely resolved, with the attenuation of HPV in response to acute hypoxia being observed inconsistently in animal studies and not conclusively observed in the only human study to test combined autonomic responses directly (Liu et al., 2007). Sympathetic pathways appear to influence HPV, consistent with their known actions on pulmonary vascular tone, with α‐adrenergic activity increasing HPV and β‐adrenergic activity attenuating HPV. This could have therapeutic benefits, as demonstrated by α‐blockade in high‐altitude pulmonary oedema, but also deleterious consequences, as with β‐blockade exacerbating HPV.

## 8 CONCLUSIONS

**FIGURE 6:**
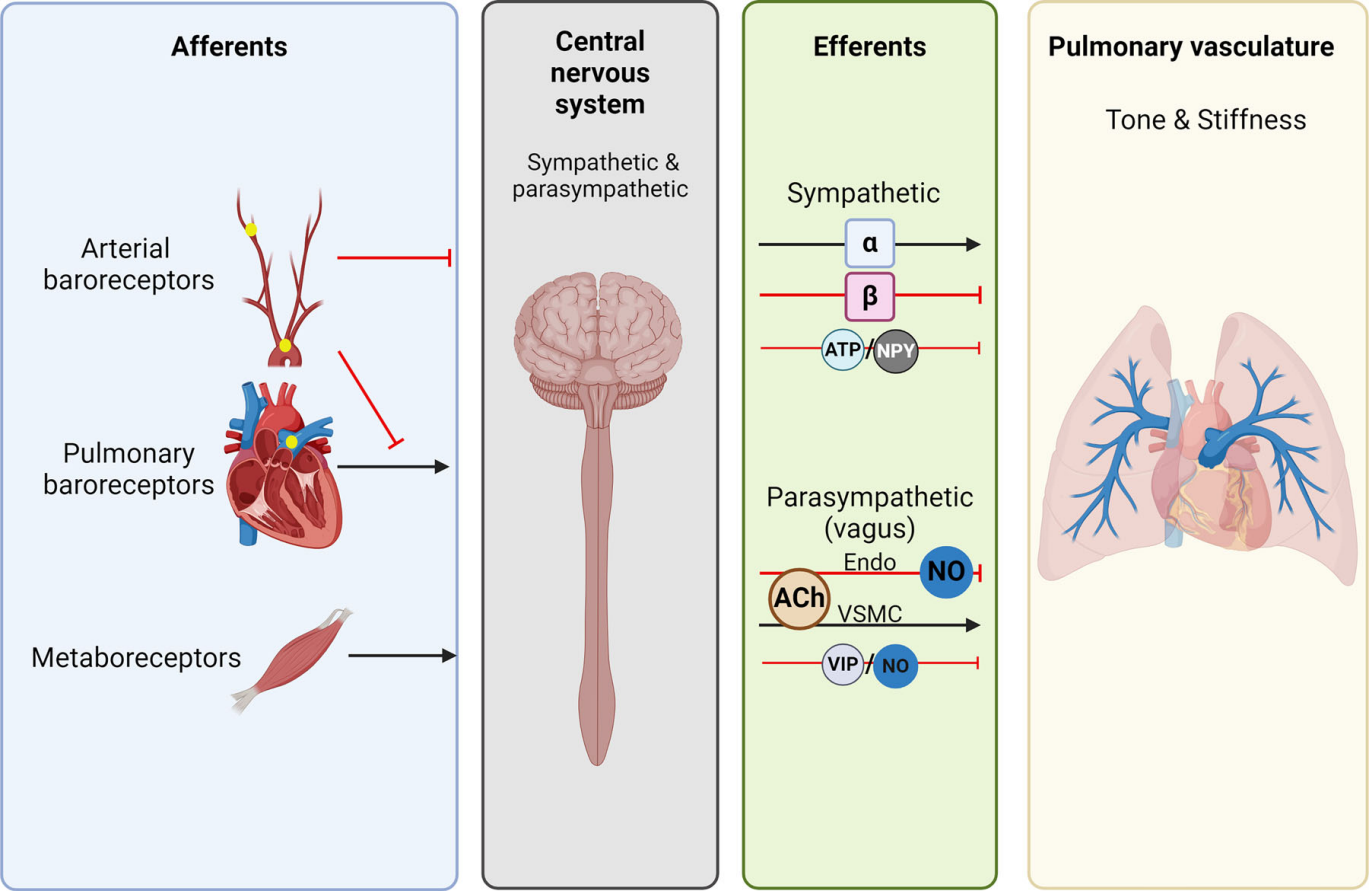
Schematic diagram summarizing autonomic neural mechanisms controlling pulmonary vascular tone. Afferent neural signals arise from pulmonary baroreceptors, systemic arterial baroreceptors and metabolically responsive skeletal muscle afferents (metaboreceptors). These have stimulatory effects (vasoconstricting; black arrows) and inhibitory effects (vasodilatory; red T‐shaped lines) on pulmonary vascular tone and stiffness. These afferent inputs are likely to be integrated in the CNS with efferent parasympathetic and sympathetic responses exerted through vagal and sympathetic efferents to the pulmonary vasculature, respectively. Sympathetic efferents increase vascular tone and stiffness via α‐adrenergic signalling (α) and reduce vascular tone and stiffness via β‐adrenergic signalling (β). ATP and neuropeptides, such as neuropeptide Y (NPY), might also be vasoconstrictive sympathetic neurotransmitters. Parasympathetic activation, predominately via cholinergic transmission (ACh), exerts dual effects of vasodilatation and reduced stiffness, mediated via the endothelium (Endo) and nitric oxide (NO), and vasoconstriction and increased stiffness, mediated via the action of vascular smooth muscle (VSMC). Vasoactive intestinal peptide (VIP) and NO also act as vasodilatory neurotransmitters for parasympathetic neurons. Created in BioRender. Plunkett, M. (2024) BioRender.com/e97z759.

Pulmonary circulation is regulated importantly by the autonomic nervous system, with key properties of vascular tone and stiffness both under the influence of sympathetic and parasympathetic nervous systems (Figure 6). The specific responses to sympathetic and parasympathetic activation are dependent on interactions with passive haemodynamic factors, mechanical effects of lung volume, local mechanisms, such as endothelium‐mediated vasodilatation or constriction, and systemic reflexes. Interactive effects with systemic homeostatic reflexes (e.g., arterial baroreflex and skeletal muscle afferents) integrate responses in the pulmonary and systemic circulation to shared stressors. However, in general, our understanding of the nature and function of these interactions in the integrated control of pulmonary circulation remains limited. These neural mechanisms can become dysregulated in PH, which has, however, led to new and promising targets in PH, including vagal potentiation (pharmacological and electrical stimulation), carotid baroreflex stimulation in preclinical animal studies, and pulmonary artery denervation in humans.

## AUTHOR CONTRIBUTIONS

Michael J. Plunkett and James P. Fisher conceptualized the paper. Michael J. Plunkett wrote the original draft and revised the manuscript. Julian F. R. Paton and James P. Fisher critically reviewed and revised the mansucript. All authors approved the final version of the manuscript. All authors agree to be accountable for all aspects of the work in ensuring that questions related to the accuracy or integrity of any part of the work are appropriately investigated and resolved. All persons designated as authors qualify for authorship, and all those who qualify for authorship are listed.

## CONFLICT OF INTEREST

The authors declare no conflicts of interest.
